# Changing Psychiatry or Changing Society? The Motion for the Rights of the “Mentally Ill” in Greece, 1980-1990

**DOI:** 10.1093/jhmas/jrab020

**Published:** 2021-08-03

**Authors:** Despo Kritsotaki

**Affiliations:** Institut national de la santé et de la recherche médicale (INSERM), Paris, France

**Keywords:** Mental patient movement, mental health, psychiatry, radical psychiatry, activism, social movements, Greece, 1970s–1980s

## Abstract

In 1980, the first formal association of mental patients, their relatives, and mental health professionals was founded in Athens, Greece. The Motion for the Rights of the “Mentally Ill” proposed a total restructuring of mental health care and a novel conceptualization of mental illness. On the one hand, it demanded that the mental health system be based on open services, psychotherapy, and on patients’ active participation in all decisions concerning their treatment and life. On the other hand, it conceptualized mental illness as a political issue that concerned all. Thus, the Motion viewed the promotion of the rights of the mentally ill as part of a broader project of cultivating conscious, active, and collective citizenship. This paper traces the Motion’s history during the 1980s, showing that it was shaped by both the socio-political conditions of Greece in the post-dictatorship period, a time of intense politicization, and by the legacy of mental patient activism in the Western world during the 1970s and 1980s. It argues that, although the Motion had a limited long-term impact, it represented the mental patient movement in Greece as it furthered the latter’s main features, most importantly its twofold endeavor to change not only the mental health system and the attitudes towards mental illness, but also society.

The Motion for the Rights of the “Mentally Ill” (henceforth the Motion) was the first formal association of mental patients in Greece. The Motion, which also included non-patients, chiefly mental health professionals and, to a lesser extent, patients’ relatives, was founded in Athens in 1980 and was formally dissolved around 2007. This paper examines the Motion’s history in the 1980s as the beginning of the mental patient movement (MPM) in Greece. Social movements are defined as “vague currents of collective sentiment” or “social forms” (groups or collectivities) that voice grievances and demand or resist change,^1^ and that have some degree of organization and continuity in space, time and activity,^2^ and a “durable culture.”^3^ As this paper will argue, the Motion did not manage to have this kind of long-lasting impact since it was a small and relatively isolated organization, one of the just two mental patient organizations in 1980s Greece. However, it can be argued that in its unique way, the Motion represented the MPM in Greece, as it introduced to the country a new approach to mental health, viewing it as a political issue and asking for broader social change. Within this context, the history of the Motion highlights important aspects of the MPM in the Western world, contributing to our understanding of the latter’s origins, ideas, activities, and legacy.^4^

In order to place the Motion within the context of the MPM in the Western world, I start by proposing an outline of the MPM, based mostly on the more researched cases of northern America and Britain. This general account concentrates on how the movement emerged within and was shaped by the critiques and innovations in mental health after the Second World War, and the broader social and cultural changes of the time. In the second part of the paper I turn to Greece, and explore when, how, and why the MPM developed there. I consider the post-war mental health reforms and socio-political situation, paying special attention to the period after the fall of the dictatorship (1974) – a relatively new field in Greek historiography.^5^ Finally, I focus on the Motion to analyze its discourses and practices, and explore how these fit in, and can further elucidate, the history of the MPM. I chose to study the first decade of the Motion’s history primarily on the basis of the rich material provided by its magazine, *Freedom Is Therapeutic*, published from 1982 to 1993. The magazine, which “echoed the concerns, climate and activities of both the Motion and the surrounding atmosphere,”^6^ along with discussions and interviews with former members, provides the main sources of this section and an entry point to the views and experiences of the Motion’s participants.^7^

## The Mental Patient Movement: An Outline

The MPM developed in the later part of the twentieth century, though earlier and with a greater intensity in some countries, such as the USA, the Netherlands and the UK, than in others, such as France, Spain and Greece.^8^ It centered around the protection of patients’ rights and most of all the right to have control over their treatment and lives. Although this paper concentrates on the first decades of the MPM, the 1970s and 1980s, it is important to note the mental health and social background that were crucial to its development, most notably the transformations of mental health care since the 1950s, and the counterculture and new social movements of the long 1960s.^9^

After the Second World War, fascism’s devastating realities reinforced the discourse of human rights, and brought isolation, separation, and torture under attack. Well-established psychiatric practices were now subjected to mounting criticism. In different countries, asylums, the main locus of psychiatry, were compared to concentration camps, and their violent and dehumanizing features were exposed, while non-psychiatric theoretical analyses brought out the repressive and social control perspective of psychiatric institutions.^10^ In some countries, non-patient advocates organized to give legal advice to patients and help them to secure their rights regarding hospital commitment and treatment.^11^ These discourses and practices were reinforced within the zeitgeist of the long 1960s. The counterculture and the social movements, including the civil rights, student, feminist, and gay rights movements, that strove for social liberalization and helped in consciousness raising, facilitated patients in evoking their identity and rights as human beings and citizens, thus giving a strong push to the MPM, as to other patient movements.^12^

Asylum-based psychiatric practice was also criticized by mental health professionals, who from the late 1940s to the 1970s developed a number of alternative theories and practices that played an important role in the rise of the MPM: the therapeutic community, institutional psychotherapy, democratic psychiatry, antipsychiatry, and radical therapy.^13^ These models were applied in different geographical and temporal contexts and in diverse ways, and had a series of differences among them, with democratic psychiatry, antipsychiatry, and radical therapy being more radical and politicized, incorporating a critique of social institutions. However, they all conceptualized a new patient role that was more active and responsible, and a new type of patient-professional relationship that was more open and equal.^14^ Certainly, the calls for a more humane and democratic psychiatric practice and the introduction of new treatments, such as group psychotherapies, did not abolish the authoritative character of psychiatry, but they helped render patient-staff relationships less rigid and hierarchical, and fostered in patients new expectations of their treatment and rights, mobilizing them to speak for themselves and demand changes. As out-patient services increased in many countries since the 1960s within the context of mental health care reforms, patients found new potential for freedom, association, and peer support. The latter proved much needed in the course of deinstitutionalization, as patients faced difficult conditions outside hospitals and struggled with stigma, poverty, and homelessness.^15^

The MPM was part of the activism of the 1970s, and sometimes even used tactics of the social movements of the time, such as demonstrations and sit-ins. Even if many groups were not directly interested in social revolution, the early MPM was politicized, as it viewed mental patients as an oppressed minority, and placed freedom in the foreground. Influenced by radical psychiatric thinking, it understood madness not as an illness but rather as an alternative state of being or a result of social conditions and oppression, while psychiatry was a harmful, repressive institution. The MPM strove to empower patients and secure their freedom of choice and the right to refuse treatment; it challenged established social and professional attitudes towards mental illness and countered negative perceptions of the mentally ill in the media. It established alternative, patient-run services, and fought to abolish involuntary hospitalization and forced treatment, especially psychosurgery and electro-convulsive therapy.^16^ Peer support and communication were important aims of the MPM, promoted not only by drop-in centers and self-help groups but also by magazines, most notably the *Madness Network News* in the USA, informal international contacts, and formal conferences, such as the National (later International) Conference on Human Rights and Psychiatric Oppression in the USA from 1973 to 1985.^17^

While the early MPM can be seen as one of the emancipatory movements of the long 1960s, in the 1980s its calls for broader social change faded, and a more moderate version of it appeared, sometimes called the consumer or user movement. This development was connected to the decline of counterculture and activism, the ebb of radical psychiatry, and the return of biological psychiatry. The consumer movement also reflected changes in mental health systems, some of which had addressed a number of the key grievances of radical psychiatry and the early MPM movement concerning involuntary treatment and patients’ rights and self-representation. The consumer version of the MPM was less critical towards the medical model of mental illness and biological psychiatry, seeing it as one of the patient-consumer’s choices in treatment, and did not discuss the issues of power, hierarchy, and coercion. Since they were less radical, consumer organizations were more easily included in decision making in health care, could collaborate with mental health professionals, and received government funding.^18^

The radical segment of the MPM, often called the survivor movement, persisted but became less prominent. It still focused on psychiatric abuse and repression, and interpreted consumers’ cooperation with professionals and officials as co-optation. Despite the early involvement of mental health professionals – mainly radical ones – in the MPM, since the mid-1970s patients were hesitant or downright opposed to the involvement of non-patients, placing emphasis on self-determination and asserting that their experiences provided expertise. Although in the 1980s some survivor groups included professionals as allies or members, they did so to a lesser extent than consumer organizations, which argued that the cooperation with professionals was necessary for the improvement of the mental health system. Therefore, throughout its history, the MPM has been characterized by ambiguities and tensions regarding the involvement of professionals, which also translated to ambiguities and tensions regarding the meaning of patients’ freedom and autonomy. If mental health care was a stable system, shaped and dominated by professionals, could patients really be free and responsible to choose how to live and be treated? Such ambiguities notwithstanding, the discourse of the MPM, both in its consumer and survivor versions, agreed on the incapacitating effects of institutionalization, and the value of choice, knowledge, self-determination, and self-advocacy, with goals to offer peer support, establish self-help alternatives, and promote recovery. Most importantly, it endorsed a new conception of patients, from isolated individuals to empowered citizens, members of a collective, who shared an identity and articulated their demands.^19^

## A Different Story: The MPM in Greece

This outline of the MPM’s course in the 1970s and 1980s is based on the cases of North America and Britain, which have been studied more extensively. In other countries, such as Greece, the MPM has been less researched, and it has been considered as slower to develop and less dynamic.^20^ Certainly, the conditions that led to the development of the MPM in other countries, namely the critiques and reforms of psychiatry from both outside and inside the mental health field, and the counterculture and social movements, were almost non-existent in Greece before the mid-1970s.

Regarding mental health care reforms, most patients were confined in asylums under inhuman conditions until the 1980s.^21^ A few islands of reform developed since the late 1940s and mainly in the 1960s and 1970s, when some mental hospitals and new extra-mural services introduced the therapeutic community, and initiated patients’ clubs, meetings, and elections.^22^ Staff members, usually younger psychiatrists, psychologists, and social workers, attempted through psychotherapy and social methods based on communication to mobilize patients and help them socialize and gain initiative and responsibility. These reforms were not radical or politicized; they did not challenge psychiatry in toto, or society. The reforms did not aim at patients’ liberation from psychiatric power and social oppression, but rather focused on their adjustment to society. Nevertheless, the reforms followed a different path from the established and authoritative psychiatry, and in the long run, they had an effect on the patients’ and professionals’ mentalities and attitudes, paving the way for a more radical approach to the rights of patients at the end of the 1970s.^23^ It is indicative that the core of the Motion for the Rights of the “Mentally Ill” were patients and professionals of one of the new services established in the early 1970s, the day care unit of the Center for Mental Health and Research in Athens. They became frustrated by the “arbitrary interventions” of the administration to the relationships between therapists and patients, when in October 1979 the director of the unit was transferred to another city.^24^ Despite the mobilization of patients and staff, the decision was not recalled and the director quit. The result was the foundation of the Motion, as well as of a new extra-mural service, the Open Psychotherapy Centre, which as we will see, was closely connected to the Motion.^25^ The case of the Motion thus corroborates that patient mobilization was cultivated in new extra-mural services, where more democratic patients-staff relationships, and new forms of treatment such as group psychotherapy, increased the expectations of the patients and enabled them to organize themselves and voice new demands.

At the end of the 1970s, the social and political conditions were ripe for such demands. In the previous decades, the post-civil-war (1946-1949) “sickly democracy”^26^ and the following military dictatorship (1967-1974) did not leave much space for counterculture and new social movements. Although these had become known in the last years of the dictatorship, and demands for social liberalization were expressed, activism concentrated primarily on political rights and democratization.^27^ Things changed after the establishment of democracy in 1974, which inaugurated a period of open expression and intense mass politicization, with strong demands for social emancipation and against discriminations and the violation of human rights.^28^ The Communist Party was legalized, political dissidents returned from exile and prisons, and the new constitution in 1975 introduced safeguards for the human dignity and the free development of personality.^29^ At the same time, in the early post-dictatorship years demands for radical social change and liberation were strengthened, as grassroots political activity exploded and movements close to the new social movements – feminist, homosexual, ecological – developed.^30^ The movements of people with physical disabilities became stronger and politicized, promoting a different concept of disability as active and empowered, and of social rehabilitation as a matter of rights and a result of struggle.^31^

In 1981, the formation of government by the socialist party, PASOK, which defined itself as a social movement for the non-privileged, and the full accession of Greece to the European Economic Community gave impetus to the process of democratization and social change with a set of social and economic measures, including policies of income redistribution, the institution of the National Health System, the reform of family law, and the redefinition of the legal position of women. By the end of the 1980s, Greek society had changed. Higher education, national health insurance, and employment in the public sector became accessible to a larger part of the population, and the stigmatization and marginalization of the Left ended. The middle classes were enlarged and social coherence improved.^32^

At this time of politicization, democratization, and concern about human rights, the inhumane and repressive character of psychiatric practice stood out. In this sense, the end of the dictatorship in Greece was an analogous historical moment to the end of the Second World War and the onset of the long 1960s in other countries: it triggered unprecedented criticism of psychiatry and induced mental health reforms.^33^ The psychiatric legislation that had been passed by the dictatorship in 1973 and 1974 was reproached for being undemocratic and not guaranteeing patients’ basic rights. After being amended in 1978, it was entirely abolished by Law 1397 of 1983 which instituted the National Health System and included mental health.^34^ The hideous conditions in the public mental hospitals, most of all of Leros, were exposed. Asylum scandals occasionally aroused the public and state’s interest in mental health care, further radicalized professionals, and mobilized the system’s reform as had happened in other countries.^35^ With or without the support of the European Economic Community, new mental health programs and services were inaugurated, including in their aims the increase of citizens’ participation in mental health care and patients’ empowerment with group psychotherapy, therapeutic communities, patients’ committees, and cooperatives.^36^ Within this context, it was not unexpected that the first two patient organizations, the Motion and the Association against Prejudice towards Mental Disorder (Continuity), were closely connected with such new mental health initiatives, namely the Open Psychotherapy Center and the professional preparation programs of the university clinic in Athens, respectively.^37^

Mental health critiques and reforms of the late 1970s and early 1980s had a radical and political dimension.^38^ Their protagonists, usually young, left-wing professionals who had studied abroad, viewed psychiatry as a locus for social intervention, and drew a parallel between the exclusion of the mentally ill and that of the communists, which had stopped only recently.^39^ They were influenced by radical psychiatric thinking,^40^ mainly by democratic psychiatry, which since the 1980s seemed particularly attractive to progressive Greek mental health professionals, and provided a model for deinstitutionalization based on the effort to restore the subjectivity and freedom of patients.^41^ The stress on the political, social, and ideological dimensions of psychiatry found a receptive audience among left-wing professionals, as well as the mentally ill and the politicized public of the post-dictatorship period. However, already by the early 1980s skepticism was expressed about the extreme appropriation of radical psychiatry. Even those who acknowledged the subjective nature of psychiatric diagnosis and treatment, the relationship of psychiatry with the state and the pharmaceutical industry, and the violations of patients’ rights, disapproved of the “pseudo-intellectuals” with their “contestation verbalisms,”^42^ which ignored the patients’ pain and need of treatment, and were therefore useless or harmful to them.^43^ The eclectic adoption of a mildly radical psychiatry was criticized by more radical thinkers as a trick of mainstream psychiatry to increase its power.^44^ However, it can be understood within the recession of radical psychiatry and activism in the 1980s in many countries, including Greece, where calls for social change were turning more moderate, and radical psychiatric critiques became less popular after the socialist party came to power in 1981.^45^ Since the late 1980s, psychiatric reform lost its political character and the orientation to social change, and it became more preoccupied with the technical and administrative aspects of the mental health system than with challenging its authoritative philosophy.^46^ Nevertheless, as the psychiatric reform progressed during the 1990s and 2000s, the issues of rights and self-representation of the mentally ill came to the foreground, with more opportunities for patients to speak and be heard. The MPM in the country was strengthened, as new patient organizations were formed and created links with international networks. While patients’ rights and participation were far from secured, by the 2000s it was recognized that progress had been made.^47^

The Motion for the Rights of the “Mentally Ill” - Demanding a Different Mental Health System

Back in the late 1970s and early 1980s, when mental patients’ rights and self-advocacy were just starting to be discussed, many professionals still considered that the condition of patients, especially of “chronic psychotics,” did not allow them to form associations, promote their rights, or ask for the best treatment for them.^48^ Τhe Motion was founded, among other reasons, to react to this view and to the zero involvement of patients in the decisions and policies that concerned them.^49^ Indicatively, in the early 1980s, during the discussions about the National Health System law, which included mental health, the Motion remonstrated that even the socialist government handed the issue to expert committees,^50^ and asked, “those who are directly interested –namely the ‘mentally ill’ – who are eaten alive by the psychiatric system, should they not be asked *finally* and participate” in the reform of the law, instead of just psychiatrists, legal professionals and other scientists?^51^

The right to participate in decision and policy making would guarantee the other rights of the mentally ill: to exist as equal citizens and independent personalities, live as they freely and consciously chose, have access to occupational rehabilitation and work, and decide if they were ill and if and how they would be treated.^52^ The Motion reported cases of rights violations, and tried to intervene by providing legal and medical advice to patients and by informing their relatives. While these efforts were not too frequent, and often not successful, the Motion demanded a complete dynamic transformation of the mental health system. It had to become less medical-centered, asylums had to close, and new legislation, not grounded on the notion of dangerousness, was needed. Further, the training of psychiatrists had to improve and the dominance of medication, which was used not for therapy but for repression, and served the profits of the pharmaceutical companies and psychiatrists, had to stop. Finally, involuntary treatment, any kind of confinement and restraint, and violent methods such as ECT had to be abolished.^53^ The reforms implemented during the 1980s did not satisfy the Motion, who saw them as simple bed increases and building renovation, while institutionalization, confinement, and repression were perpetuated. It criticized the law of 1983 for introducing only minor changes to the psychiatric legislation of the dictatorship, and for discriminating against the mentally ill, not protecting their rights, and depriving them of the control over their treatment.^54^

## Disseminating “Another Truth on Psychiatric Issues”

For the Motion, the existing mental health system, with its involuntary and violent “treatments,” but also the media, with their overstatements on the dangerousness of the mentally ill, criminalized mental illness and propagated ignorance and stigma.^55^ The Motion aimed at changing professional and lay approaches to mental illness through dissemination and publicization activities, such as interviews in magazines and newspapers, radio broadcasts, presentations or interventions in professional conferences, and public events. Most notably, in 1984 and 1985 the Motion organized two public events in two neighborhoods of Athens, and in 1983 and 1984 participated in the festival Avgis-Thouriou, a yearly political and cultural event organized since 1975 by the Eurocommunist youth organization of Rigas Ferraios, which was affiliated with the Communist Party of Greece of the Interior.^56^ Various organizations, including the gay rights movement organization AKOE, were taking part in the festival, a fact that brings out the openness of the Communist Party of Greece of the Interior toward social movements. The Motion’s participation in the festival offers a hint of its political inclination to the Left and particularly to the New Left. This political orientation went hand in hand with the Motion’s agenda on human rights and broader social change, with the emphasis, as we will see, on active citizenship and pluralism.

An important communication vehicle was the Motion’s magazine, *Freedom Is Therapeutic*, with ten issues from 1982 to 1993. The magazine presented the Motion’s views and activities, included information on different treatments and mental health systems, and published reviews of books, magazines and films, poems and prose, anonymous or eponymous, Greek and translated, humorous texts and caricatures. The magazine’s foremost function was to further the Motion’s aim to break the silence and make the voices of the mentally ill heard, which was a first step for their self-determination and inclusion in all decisions that concerned them. To this end, the Motion asked readers to write about their experiences and complaints. Still, especially in the first issues, many articles were anonymous, which can be interpreted as a sign of collective redaction, but also as an indication of the authors’ reluctance to reveal their names because of the stigma and the “internalized censorship and repression.”^57^

In total, the Motion strove to show “another truth on psychiatric issues” as issues that concerned everyone, since mental illness was seen not a fixed or objective category. As the quotation marks in the phrase “Mentally Ill” in the title of the Motion implied, mental illness was viewed less as a disease and more as a label that the family, society, and state used for those who were different from the statistical norms, including homosexuals, drug addicts, anarchists, activists, the voluntarily unemployed, or simply those with an independent behavior.^58^ The Motion located the root of the problem in society and its institutions. It argued, in an anti-psychiatric vain, that modern society exterminated joy, desire, imagination, and laziness, thus making people crazy, and along with the family, turned its most vulnerable members into scapegoats.^59^ “In a society that is ill, we are all possible ‘patients,’” claimed the Motion,^60^ similarly to patient organizations in other countries.^61^ “Tomorrow, our brother or us” could be inmates of the psychiatric hospital of Leros, and thus it was necessary for everyone to stop being indifferent and fight, wrote a member of the Motion in 1983.^62^ Psychiatry was regarded more as part of, rather than solution to, the problem. Along with the media, it assisted the family, society, and the state in safeguarding normality, excluding and humiliating those who did not fit in. Psychiatrists substituted for policemen, and mental hospitals – from public asylums to private clinics – served not the patients but their families, communities, and the state, and exterminated the inmates’ individuality, personality, imagination, even physical existence. Moreover, mental health sciences were constantly expanding their control, in a process of “psychiatrization” of everyday life, which was part of the “everyday fascism” against those who were different.^63^

## Combining Radical and Moderate Approaches

The above discourse incorporated many elements of radical thinking about psychiatry, while the Motion’s magazine featured texts of and about Thomas Szasz, R. D. Laing, Michel Foucault, Félix Guattari, the German Socialist Patients Collective, and democratic psychiatry. Some of these elements were being discussed in other alternative mental health agents, such as reformed services, but certainly not in the majority of mainstream mental health institutions. Thus the Motion was in the vanguard of radical thinking about mental health care in 1980s-Greece. In particular, though democratic psychiatry was not without shortcomings, its impressive results in the neighboring country exercised the strongest and most lasting influence on the Motion.^64^ Indicatively, a catch phrase of the Motion and the title of its magazine came from the slogan of democratic psychiatry, “freedom is therapeutic.”^65^ The radical elements of the Motion were particularly potent in the early 1980s, a time of “night meetings, endless conversations, a feeling of revolution and contestation, punch to the eye of authority.”^66^ However, even at this time the Motion’s radicalism was combined with a more moderate discourse, which became stronger in the later part of the 1980s, even though the Motion remained highly critical of the repressive social and political functions of psychiatry. Within the context of the national and international decline of radical psychiatric thinking during the 1980s, the Motion never negated the existence of mental illness, and it disapproved of “strange views,” such as that were no mental problems but only organic brain disturbances, that mental illness was caused by capitalist society, or that it could/should not be treated.^67^ On the contrary, it accepted mental health professional approaches, even medication, as long as they were applied in a humane and individualized way, based on social psychiatry and psychotherapy, and undertaken in extra-mural services with the individual’s free will.^68^

The interweaving of heterogenous influences and perceptions, which led to a rather eclectic and ambivalent outlook, was also reflected in the relationship between the Motion and mental health professionals. The latter were among the founding members of the Motion, and continued being members and presidents, playing an important part in representing it in public and writing in its magazine. Although they participated in equal numbers with non-professionals, they constituted a more stable segment of the Motion, and were the ones to encourage patients and relatives to join, even more so at times when the number of non-professionals decreased.^69^ This involvement of professionals might seem as contradictory to the Motion’s position against the “passivitization and submission” of the patients, and for their liberation “from the constant and exclusive guidance of experts,” in order to take their fate into their hands.^70^ The Motion was particularly critical towards psychiatrists in positions of authority, such as university professors and public hospitals directors, who were deemed accountable for the repressive and violent mental health system in Greece.^71^ The Motion often reproached even professionals who initiated new types of community and social psychiatry services for not taking a stance against the violation of human rights and the use of psychiatry as a tool of discipline and control.^72^

The Motion’s ambivalent stance towards professionals was to a degree balanced out by the fact that its professionals were not part of the academic or public psychiatric establishment, but rather alternative professionals, usually young psychiatrists and in many cases psychologists, occupational therapists, psychotherapists, and social workers. Most of them were working in the Open Psychotherapy Center, where also most of the patient members were or had been under treatment.^73^ Indeed, the Center can be seen as the major enlisting structure for the Motion. The two institutions, although distinct, were closely allied. They had, as noted above, a common beginning, and they also had common views regarding stigma, the rights of the mentally ill, and the restructuring of the mental health system on the basis of open services and psychotherapy.^74^ They organized common activities, such as discussions and talks within the frame of Center’s seminars, while the Motion’s magazine published advertisements and presentations of the Center. Although in the later part of the 1980s the Motion tried to differentiate itself from the Center, the connections remained.^75^ This indicates the difficulties of a patient organization to function without the support of a professional structure.^76^ Cooperation with professionals seems to have been the only means to build a MPM in Greece, and the international context reinforced this tendency, as the MPM’s radicalism and separatism (the exclusion of professionals from patient organizations) was receding.

The ambivalence towards professionals was, as already mentioned, a universal aspect of the MPM. In the case of the Motion, another point of ambiguity related to its contacts with foreign mental health organizations. By exchanging information and ideas with both radical and moderate foreign organizations, and drawing material from their publications,^77^ the Motion was endeavoring to belong to and get support from an international network, regardless of its precise orientation. Probably sensing the unifying elements of the different segments of the MPM that I underlined in the first section of this paper, the Motion stressed the importance of the awareness that the problem of mental illness was common for all humans and that, all over the world, people thought and fought as the Motion did in Greece.^78^ Eventually, as the Motion’s moderate side became more prominent and as more opportunities for international networking were given within a moderate framework, the Motion participated in the First European Conference of Users of Mental Health in the Netherlands in 1991. The conference was sponsored by the European Economic Community, the World Federation of Mental Health, and the Dutch Ministry of Health, hosted organizations from nineteen countries, and led to the establishment of a European network, which in 1998 became the European Network for (ex)-Users and Survivors of Psychiatry.^79^

## Being Political

Even if the Motion combined moderate with radical perceptions and demands, it approached mental illness and its handling in a radically new way: as political.^80^ Inspired by democratic psychiatry and the idea that all psychiatric practices establish a political relationship between the patient and society,^81^ the Motion presented itself as the first politicized activity of the mentally ill,^82^ and as “a movement of social contestation.”^83^ Founded by patients, mental health professionals, and other people “interested in the social and political aspect of the issue mental health,” it viewed the lack of concern about the constant violation of the rights of the mentally ill as a sign of weakened political consciousness.^84^ It asserted, “To the extent to which we don’t have full consciousness of the ‘therapy’ process that they impose on us, and we don’t actively react against it, we are politically dead.”^85^ Moreover, since anyone was a potential victim of stigmatization and repression with the help of psychiatry, particularly as everyday life was becoming more “psychiatrized,” the fight for self-determination had to be collective. In other words, a broader, politicized anti-asylum movement was necessary. It would be based on those subjected to the consequences of the asylum, namely patients and nurses, and it would be framed by wider social and political forces who wanted social change.^86^

The specific situation in Greece in the 1980s – especially the early 1980s – was the necessary background that shaped and allowed to emerge this new understanding of mental illness and health care. The cultivation of conscious, active, and collective citizenship was important during the post-dictatorship period, if the rise of authoritarianism in the future was to be prevented. What was more, as was the case for several progressive and radical voices of the time, the Motion did not regard the end of the dictatorship as the end of political and social repression.^87^ It denounced “the authority’s violence against the autonomous development and self-determination of the personality” and called for the continuation of the struggle that students had begun in 1973, which it interpreted as a fight not only against political authoritarianism but also more generally for social liberation.^88^ It denounced not only psychiatric but also police violence, extensive in the late 1970s and early 1980s, especially in Exarchia, the neighborhood of Athens where the Motion’s office was located, and a key area for anarchist and autonomist groups.^89^ Worrying that police violence could easily expand to more and more citizens, the Motion was solidary to all “minorities,” from anarchists and prisoners to homosexuals and drug addicts, as they all fought for their rights and against their stigmatization, criminalization, humiliation, and persecution.^90^ Within this context, the Motion published in every issue of *Freedom Is Therapeutic* an open call to all interested to attend its weekly meetings, and endeavored to establish a “broad collaboration” with “progressive individuals” and “social movements.” It had contacts and cooperation, participating in common events and co-authoring texts, with various organizations and groups, including the Liberation Movement of Homosexuals of Greece (AKOE), the Group of Prisons, the Committee for the Right Information and Handling of Drugs, and the Alternative Ecological Motion.^91^

The Motion did attract the interest of a non-patient and non-professional audience, especially young people who were drawn to the anarchist, feminist, gay and/or anti-psychiatric discourses, frequented Exarchia, and read the magazine of the Motion, which was sold in the kiosks and the anarchist bookshop of the area. However, this audience was not prepared to become actively involved in the Motion’s work. Their interest was less connected to the rights and problems of the mentally ill than to the meanings of madness in relation to the self and the revolt against the dominant social and political system and culture. In addition, they did not know exactly who partook in the Motion, and felt uneasy towards it; mental illness remained difficult to talk about or connect to and it was still surrounded by fear and stigma. On the contrary, other organizations of the time, for example feminist and gay, were more accessible as their members were more recognizable in the public sphere and their narratives appealed to broader audiences.^92^ It is indicative that, although the Motion was optimistic about its participation in the festival Avgis-Thouriou and aspired in future collaborations with other parties and organizations, this practice did not seem to continue after 1984.^93^ The lack of a wider interest in the issues of the mentally ill, at least in the way that the Motion understood them, was also evidenced by the fact that newspapers usually did not publish the denunciation texts that the Motion sent them.^94^

The Motion faced difficulties in extending its reach not only beyond the mental health field, but also within it. It was a small organization, not only in quantitative terms of members and activities – which was a usual feature of the mental patient organizations in many countries – but also in terms of the momentum that would further the MPM in Greece. Moreover, in the late 1980s and the beginning of the 1990s, it lost many of its members, especially patients, and its activities weakened. Significantly, the last issue of the magazine was published in 1993, four years after the previous one. In addition, since the mid-1980s as the Motion became less radical, it also became less political and polemic. It turned to new goals, such as international collaborations and the collection of data about mental health institutions in Greece.^95^ Its magazine published more varied material and fewer denunciations and political statements.^96^ The loss of dynamism and political orientation was linked to the gradual weakening of collective activity and politicization in Greece since the mid- and late 1980s, but also to the shift of the psychiatric reform movement in the country in a less political and ideological direction.^97^ Or, as the Motion lamented in 1988, psychiatric reform had failed in Greece before it had even started, as it had been deprived of its social and political dimensions and had been reduced to the object of a guild of mental health experts, predominantly psychiatrists.^98^ Within this context, mental patient organizations in the 1990s were more orientated towards professional rehabilitation and artistic expression, and less concerned with larger social issues such as the psychiatrization of life and the “everyday fascism” against those deemed different. They were less politicized and did not aim at forming or participating in a broad social movement against authoritarianism and for social liberation, but centered more on the specific rights of mental patients, their inclusion in decision making, the fight against stigma, and self-help. The Motion seems to have followed this trend in the early 1990s, when it enlisted new members, mainly through the Open Psychotherapy Center, and managed to continue its meetings, organize some events, and initiate a new self-help group until it was officially terminated around 2007.^99^

## Conclusions

Mental health critiques and reforms in the Western world during the second part of the twentieth century together with the activism of the long 1960s paved the way to a more active patient role and a less hierarchical and more democratic types of relationships between patients and professionals. All this contributed to the emergence of the MPM, which had a varied course and impact in different countries. In Greece, the MPM appeared at a later time – the first formal association of mental patients was founded in 1980 – and its presence was less marked. However, it shared many of the key features of MPM organizations, which it can help further highlight.

An important common aspect of the Motion and the MPM was their double origin in psychiatric reforms and social activism, which corresponded to their double goal, to change not only the mental health system and the attitudes towards mental illness, but also society. This aspiration was shaped mainly by leftist-leaning ideologies, the social movements, and the radical psychiatric thinking of the long 1960s, and in Greece of the late 1970s and early 1980s. In this framework, mental illness and mental health were understood as social and political issues inextricably linked to the efforts to promote political consciousness, democracy, tolerance, and social liberation. The linking of mental health with socio-political demands was a novel and significant element of both the early MPM and the Motion. However, a broad mobilization around mental illness was hard to achieve even by developed examples of the MPM in other countries, especially as activism and politicization ebbed in the 1980s, and indifference and prejudice around mental illness persisted. So, it should not be surprising that “almost none of the positions and the goals of the [M]otion materialized in the mental health field.”^100^ The mission to establish mental illness and health as political issues that concerned everyone in the framework of a broader social movement, and to remove mental health care and reform from the sole authority of psychiatrists and administrators, proved unattainable.

Nevertheless, the Motion was not without accomplishments. It promoted a novel discourse on mental illness in Greece at a time when public narratives on the subject were centering on philanthropy, hospital scandals, and the dangerousness of the mentally ill. It disseminated information, exposed abuses, and furnished a network of communication and support, helping at least some of the mentally ill to counter the fear, stigma, and shame, and claim the title of the expert of their health and life.^101^ Belonging to the Motion legitimized the members’ experiences, and challenged established perceptions of mental illness and mental health care. Therefore, the Motion shared the MPM’s major themes, aims, and activities: the establishment of contacts and communication; the spread of information; the breaking of isolation and silence, and the publicization of mental illness issues. All this was enabled by the Motion’s (and the MPM’s) tendency to blur the boundaries between mental illness and health, and perceive them as socio-political matters. In other words, the Motion shared and promoted two main aspects of the mental (and other) patients’ movements: it strove to turn (mental) illness from a personal to a public issue, and to promote a new notion of citizenship, based not only on responsibilities and rights, but also on collective action and solidarity.^102^ This was an important legacy that even small MPM organizations with limited impact such as the Motion managed to leave behind.

## Funding

This project has received funding from the European Union’s Horizon 2020 research and innovation programme under the Marie Sklodowska-Curie grant agreement No 793875.

## Footnotes

1 Mayer Zald and John McCarthy, *Social Movements in an Organisational Society* (New Jersey: Transaction, 1994), cited in Nick Crossley, *Contesting Psychiatry: Social Movements in Mental Health* (London: Routledge, 2006), 11; Brigitte Chamak, “Autism and Social Movements: French Parents’ Associations and International Autistic Individuals’ Organisations,” *Sociology of Health & Illness* 30 (2008): 76–96.

2 David Snow, Sarah Soule, and Hanspeter Kriesi, “Mapping the Terrain,” in *The Blackwell Companion to Social Movements*, ed. David A. Snow and Sarah A. Soule (New York: John Wiley & Sons, Ltd, 2007), 3–16.

3 Nick Crossley, “Fish, Field, Habitus and Madness: The First Wave Mental Health Users Movement in Great Britain,” *British Journal of Sociology* 50 (1999): 647–670.

4 Since this is the first thorough historical study of a mental patient organization in Greece, the paper cannot ignore the western framework, which served as a major inspiration for historical actors, and also offers the main historiographical frame today. At a further point of the research, it would be useful to place the Greek case within a broader international context, including the European South and Latin America, especially as they were countries where, similarly to Greece, military regimes were established. See for example, Rafael Huertas, “Psychiatrists and Mental Health Activism during the Final Phase of the Franco Regime and the Democratic Transition,” *History of Psychiatry* 30 (2019): 77–89; Cristian R. Montenegro and Flora Cornish, “Historicising Involvement: The Visibility of User Groups in the Modernisation of the Chilean Mental Health System,” *Critical Public Health* 29 (2017): 61–73.

5 For a sample of recent work on post-dictatorship Greece period, see Manos Avgeridis, Efi Gazi, and Kostis Kornetis, ed., *Μεταπολίτευση. Η Ελλάδα στο μεταίχμιο δύο αιώνων [The Post-Dictatorship Period. Greece between Two Centuries]* (Athens: Themelio, 2015).

6 Natasa Karapostoli, “‘Αναμνηστικόν’. Η Κίνηση για τα Δικαιώματα των Ψυχασθενών [’Anamnesis’. The Motion for the Rights of the Mentally Ill],” *Αντιστίγμα. Δίκτυο για τα Δικαιώματα και τις Παραβιάσεις στο Χώρο της Ψυχικής Υγείας [Anti-Stigma. Network for the Rights and the Violations in the Field of Mental Health]*, no. 3 (2009): 12–13, 13.

7 Judi Chamberlin, a leading figure of the MPM, emphatically stated in 1999 that, “Our history will be recorded and written by *us*.” Chamberlin reflected the need for patient self-determination and representation. Linda Morrison, *Talking Back to Psychiatry: The Psychiatric Consumer/Survivor/Ex-Patient Movement* (Oxon: Taylor & Francis, 2009), 57. Although this is an important and central demand of the MPM, the history of the movement is and should be understood as part of social and cultural history on the national and international level. Thus, not being a patient and/or a participant in the movement should not be an obstacle to studying it.

8 Some antecedents of the MPM have been traced back in the nineteenth and early twentieth centuries, for example the Alleged Lunatics’ Friends Society, founded in 1845, the campaigns of Elisabeth Packard in the 1860s and 1870s, and the mental hygiene movement in the early twentieth century. See Mohammed Abouelleil Rashed, *Madness and the Demand for Recognition: A Philosophical Inquiry into Identity and Mental Health Activism* (Oxford: Oxford University Press, 2019), 5–7. These initiatives, however, were isolated before the 1970s, when the MPM gained momentum.

9 The long 1960s cover the period from the late 1950s to the mid-1970s. Arthur Marwick, *The Sixties: Cultural Revolution in Britain, France, Italy, and the United States, c.1958-c.1974* (Oxford: Oxford University Press, 1998). New social movements have been defined as those that center less on social class and the means and organization of production and more on a common identity, including personal and cultural aspects of life, which they turn to political and public issues. The term is usually used for the civil rights, women’s liberation, and gay rights movements, but the distinction between old and new social movements has been contested. Alain Touraine, “An Introduction to the Study of Social Movements,” *Social Research* 52 (1985): 749–587; Vololona Rabeharisoa, “From Representation to Mediation: The Shaping of Collective Mobilization on Muscular Dystrophy in France,” *Social Science & Medicine* 62 (2006): 564–576; Tom Shakespeare, “Disabled People’s Self-Organisation: A New Social Movement?” *Disability, Handicap & Society* 8 (1993): 249–264. Counterculture is also an ambiguous term, which, for the purposes of this research, is understood as “an ongoing project of self-conscious cultural producers who tried to build more autonomy into their lives and who did so with a sense that they were working not as lone practitioners…, but as part of a collective experiment in community building.” David Farber, “Building the Counterculture, Creating Right Livelihoods: The Counterculture at Work,” *The Sixties* 6 (2013): 1-24, 3.

10 An early and famous exposé was Albert Deutsch, *The Shame of the States* (New York: Harcourt, Brace and Company, 1948), while key early examples of non-psychiatric analyses included Erving Goffman, *Asylums: Essays on the Social Situation of Mental Patients and Other Inmates* (New York: Anchor Books, 1961); Michel Foucault, *Histoire de la folie à l’âge classique* (Paris: Plon, 1961).

11 Nick Crossley, “The Field of Psychiatric Contention in the UK, 1960–2000,” *Social Science & Medicine* 62 (2006): 552–563; Phil Brown, “The Mental Patients’ Rights Movement and Mental Health Institutional Change,” *International Journal of Health Services* 11 (1981): 523–540; Alexander Dunst, “‘All the Fits That’s News to Print’: Deinstitutionalisation and Anti-Psychiatric Movement Magazines in the United States, 1970–1986,” in *Deinstitutionalisation and After: Post-War Psychiatry in the Western World*, ed. Despo Kritsotaki, Vicky Long, and Matthew Smith (Cham: Springer International Publishing, 2016), 57–74.

12 Organizations of patients suffering mainly from chronic diseases and disabilities had started to appear in the 1940s, but since the mid-1960s they began to place more emphasis on patients’ autonomy, rights, and responsibilities. For patient organization movements or health social movements, see the special issues of *Sociology of Health and Illness* 26, no. 6 (2004) and *Social Science & Medicine* 62, no. 3 (2006). In the same period, a more general trend in health care emerged, calling for the contribution of users to the organization and implementation of their health care. David Rothman, *Strangers at the Bedside: A History of How Law and Bioethics Transformed Medical Decision Making* (New York: Basic Books, 1991).

13 While innovations had begun before the Second World War, and while not all of post-war psychiatry was reformist or revolutionary, after 1945 the spirit of change became more widespread than before. Nicolas Henckes, “Reforming Psychiatric Institutions in the Mid-Twentieth Century: A Framework for Analysis,” *History of Psychiatry* 22 (2011): 164–181; Isabelle von Bueltzingsloewen, “Le militantisme en psychiatrie, de la Libération à nos jours. Quelle histoire ?!” *Sud/Nord* 25 (2010): 13–26.

14 Catherine Fussinger, “Eléments pour une histoire de la communauté thérapeutique dans la psychiatrie occidentale de la seconde moitié du 20e siècle,” *Gesnerus* 67 (2010): 217-540; Marquet Jérémie, “Contribution à un certain mouvement vers une psychothérapie institutionnelle: entre pérennité fragile et pérenne fragilité” (Université du droit et de la santé, Lille, 2013); John Foot, *The Man Who Closed the Asylums: Franco Basaglia and the Revolution in Mental Health Care* (London: Verso, 2015); Oisín Wall, *The British Anti-Psychiatrists: From Institutional Psychiatry to the Counter-Culture, 1960-1971* (London: Routledge, 2018); Dunst, “‘All the Fits That’s News to Print.’” The term antipsychiatry was introduced by David Cooper, one of the protagonists of the movement, but was refuted by others, most notably by R.D. Laing, while it is rejected by some scholars today. However, the term has been established to describe a series of radical critiques and alternative practices between the mid-1960s and mid-1970s. For a comparison of antipsychiatry with institutional therapy and democratic psychiatry see, Catherine Fussinger, “‘Therapeutic Community,’ Psychiatry’s Reformers and Antipsychiatrists: Reconsidering Changes in the Field of Psychiatry after World War II,” *History of Psychiatry* 22 (2011): 146–163; Oisín Wall, “Basaglia and the British Anti-Psychiatrists, 1960–70,” in *Basaglia’s International Legacy: From Asylum to Community*, ed. Tom Burns and John Foot (Oxford: Oxford University Press, 2020), 23–42. The fluidity and interaction between these different models allows their grouping under a broad category of alternative and radical psychiatry.

15 Sue Estroff, *Making It Crazy: An Ethnography of Psychiatric Clients in an American Community* (Oakland: University of California Press, 1985); Dunst, “‘All the Fits That’s News to Print.’”

16 Brown, "The Mental Patients’ Rights Movement"; Dunst, "‘All the Fits That’s News to Print'"; Crossley, "Fish, Field, Habitus and Madness"; Athena Helen McLean, “From Ex-Patient Alternatives to Consumer Options: Consequences of Consumerism for Psychiatric Consumers and the Ex-Patient Movement,” *International Journal of Health Services* 30 (2000): 821–847.

17 Dunst, “‘All the Fits That’s News to Print.’” Since the 1980s, international meetings and conferences increased, and since the 1990s the international networking and coordination of the MPM became stronger. http://enusp.org/who-we-are/; http://www.wnusp.net/index.php/history-of-the-world-network-of-users-and-survivors-of-psychiatry.html.

18 Morrison, *Talking Back to Psychiatry*, 57–87; McLean, “From Ex-Patient Alternatives to Consumer Options.”

19 McLean, "From Ex-Patient Alternatives to Consumer Options"; Anne Rogers and David Pilgrim, “‘Pulling down Churches’: Accounting for the British Mental Health Users’ Movement,” *Sociology of Health & Illness* 13 (2008): 129–148.

20 Center of European Constitutional Law – Themistoklis & Dimitris Tsatsos Foundation and National Federation of People with Disability, *Ανάπτυξη και ρόλος του κινήματος αυτoσυνηγορίας σε άλλες χώρες [Development and Role of the Movement of Self-Advocacy in other countries]* (Athens, 2012), 172.

21 In the late 1970s there were about 10,000 inmates in the public psychiatric hospitals of the country. Kostas Mpairaktaris, “Αξιολόγηση της δημόσιας ιδρυματικής περίθαλψης στην Ελλάδα [Assessment of the Public Psychiatric Care in Greece],” in *Ελληνογαλλικό Συμπόσιο Κοινωνικής Ψυχιατρικής [Greek-French Symposium of Social Psychiatry]*, ed. Serge Lebovici and Panagiotis Sakellaropoulos, vol. 2 (Athens: Kastaniotis, 1989), 59-81. At the beginning of the next decade, most of the inmates were chronic, middle-aged patients, who were involuntarily hospitalized for long periods. Michalis Madianos, *Η Ψυχιατρική Μεταρρύθμιση και η Ανάπτυξή της από τη Θεωρία στην Πράξη [The Psychiatric Reform and its Development in Theory and Practice]* (Athens: Ellinika Grammata, 1994), 194.

22 Maria Fafaliou, *Ιερά Οδός 343. Μαρτυρίες από το Δρομοκαíτειο [Iera Odos 343. Testimonies from Dromokaitio]* (Athens: Kedros, 1995), 267–270. The term “islands of reform” (from W. Rudloff, “Rehabilitation und Hilfen für Behinderte,” cited in Christof Beyer, “‘Islands of Reform’: Early Transformation of the Mental Health Service in Lower Saxony, Germany in the 1960s,” in *Deinstitutionalisation and After*, 99–114) is suited to the Greek case, as the reforms were applied on a limited level, and coexisted even within the same mental hospitals with deplorable conditions, restraint, and violence. The new methods varied from the novel physical methods of the time (ECT, leucotomies, and the new drugs from the mid-1950s) to social methods and psychotherapies (occupational therapy, sport and social activities, limitation of restraint, group psychotherapies, and psychodrama) and outpatient care. Despo Kritsotaki and Dimitris Ploumpidis, “Progressive Science Meets Indifferent State? Revisiting Mental Health Care Reform in Post-War Greece (1950-1980),” *Dynamis: Acta Hispanica Ad Medicinae Scientiarumque Historiam Illustrandam* 39 (2019): 99-121.

23 Georgios Lyketsos, a psychiatrist who initiated many of these reforms since the 1950s, later described them as “taming” the patients. The moderate character of the changes was highlighted by a younger psychiatrist, Marios Markidis, who noted that Lyketsos was not an anti-psychiatrist, but similarly to them he had the ability to go out of the common cynical mind of the “down to earth,” domineering psychiatrists. Georgios Lyketsos, *Το μυθιστόρημα της ζωής μου [The Novel of my Life]* (Athens: Gavriilidis, 1998), 268, 280.

24 Rena Hatzidaki, “Αντιμετώπιση ‘ψυχασθενών’: θεσμοί και νοοτροπίες [Handling the ‘Mentally Ill’: Institutions and Mentalities],” *Η ελευθερία είναι θεραπευτική [Freedom is Therapeutic]*, no. 2 (1983): 3-8, 3.

25 In other countries too, patient organizations were sometimes formed when the function of a psychiatric service was threatened. See for example, the case of Paddington Day Hospital and the Mental Patients Union in the UK. Crossley, *Contesting Psychiatry*, 128.

26 Ilias Nikolakopoulos, *Η καχεκτική δημοκρατία. Κόμματα και εκλογές, 1946–1967 [The Sickly Democracy. Parties and Elections, 1946–1967]* (Athens: Patakis, 2001).

27 Nikos Serdedakis, “Συνέχειες και ασυνέχειες της συλλογικής δράσης κατά τη μετάβαση από την ‘καχεκτική δημοκρατία’ στη ‘μεταπολίτευση,’ [Continuities and Discontinuities of Collective Action during the Transition from the ‘Sickly Democracy’ to the ‘Post-Dictatorship Period’]” in *The Post-Dictatorship Period*, 99–115; Kostis Kornetis, *Τα παιδιά της δικτατορίας: φοιτητική αντίσταση, πολιτισμικές πολιτικές και η μακρά δεκαετία του εξήντα στην Ελλάδα [Children of the Dictatorship: Student Resistance, Cultural Politics and the Long ’60s in Greece]*, trans. Pelagia Marketou (Athens: Polis, 2015), 28–31, 627, 636; Nikolaos Papadogiannis, “From Coherence to Fragments: ‘1968’ and the Making of Youth Politicisation in Greece in the 1970s,” *Historein* 9 (2009): 76–92.

28 Yannis Voulgaris, *Η Ελλάδα της μεταπολίτευσης, 1974–1990 [Post-Dictatorship Greece, 1974–1990]* (Athens: Themelio, 2002), 17, 20, 54, 43–47.

29 Vangelis Karamanolakis, *Ανεπιθύμητο παρελθόν. Οι φάκελοι κοινωνικών φρονημάτων στον 20ό αιώνα και η καταστροφή τους [Undesirable Past. The Files of Social Beliefs in the 20th Century and their Destruction]* (Athens: Themelio, 2019), 129, 161–166; A. Ziakas, “Η άσκηση της ψυχιατρικής στην Ελλάδα από νομική άποψη [The Exercise of Psychiatry in Greece from the Legal Perspective],” *Freedom Is Therapeutic*, no. 2 (1983): 25–27.

30 Kornetis, *Children of the Dictatorship*, 579, 592–595; Kostis Kornetis, “Μεταβάσεις, συλλογική μνήμη και δημόσια ιστορία στην Ελλάδα, την Ισπανία και την Πορτογαλία [Transitions, Collective Memory and Public History in Greece, Spain and Portugal],” in *The Post-Dictatorship Period*, 393–414; Dimitris Glystras, “Η ‘άλλη’ αριστερά. Πολιτισμική συγκρότηση και πολιτικά υποκείμενα (1974-1981) [The ‘Other’ Left. Cultural Formation and Political Subjects (1974-1981)]” (University of Athens, Department of History and Archaeology, 2017), 24.

31 Petros Karavasilis, interview by the Archives of Contemporary Social History (ASKI), http://metapolitefsi.com/%CE%A3%CF%85%CE%BD%CE%B5%CE%BD%CF%84%CE%B5%CF%8D%CE%BE%CE%B5%CE%B9%CF%82#video1; Aigli Chatzouli, *Θαλασσαιμικές ζωές: βιολογική διαφορά, κανονικότητα, βιοκοινωνικότητα. Μια ανθρωπολογική προσέγγιση [Thalassaemic Lives: Biological Difference, Normalcy, Biosociality. An Anthropological Approach]* (Athens: Patakis, 2012), 55–57.

32 Vasilis Vamvakas and Panagis Panagiotopoulos, “Η Ελλάδα στη δεκαετία του ’80. Κοινωνικός εκσυγχρονισμός, πολιτικός αρχασμός, πολιτισμικός πλουραλισμός [Greece in the 1980s. Social Modernization, Political Archaism, Cultural Pluralism],” in *Η Ελλάδα στη δεκαετία του ’80. Κοινωνικό, πολιτικό και πολιτισμικό λεξικό [Greece in the 1980s. Social, Political and Cultural Dictionary]*, ed. Vasilis Vamvakas and Panagis Panagiotopoulos (Athens: Epikentro, 2014), ΧΧΧΙ-LXXI; Papadogiannis, “From Coherence to Fragments;” Karamanolakis, *Undesirable Past*, 282, 125, 263–264, 239; Konstantina Mpotsiou, “Ν.Δ. ΚΑΙ ΠΑΣΟΚ, 1974-1985: Η ‘Ευρώπη’ ως πολιτική και ως ταυτότητα [N.D. and PASOK, 1974-1985: ‘Europe’ as Policy and Identity],” in *The Post-Dictatorship Period*, 211–224.

33 This analogy does not mean to obscure the differences between the 1950s and long 1960s on the one hand, and the late 1970s and 1980s on the other. It aims, nonetheless, to highlight that during these different eras, a similar spirit of mental health care reform and challenge of psychiatry characterized the Western world and Greece respectively.

34 Kritsotaki and Ploumpidis, “Progressive Science Meets Indifferent State?”; Maria Mitrosyli, *Ψυχιατρείο Λέρου και μεταρρύθμιση. Δημόσιες πολιτικές, ίδρυμα, ασθενείς, κοινότητα [The Mental Hospital of Leros and Reform. Public Policies, Institution, Patients, Community]* (Athens: Papazisis, 2015), chap. 2.

35 Brown, "The Mental Patients’ Rights Movement"; Jonathan Toms, “Citizenship and Learning Disabled People: The Mental Health Charity MIND’s 1970s Campaign in Historical Context,” *Medical History* 61 (2017): 481–499.

36 Rena Hatzidaki, “Ψυχιατρική ‘περίθαλψη’ και ψυχιατρική ‘μεταρρύθμιση’ στην Ελλάδα: το έγκλημα με τη φορεσιά της αθωότητας [Psychiatric ‘Care’ and Psychiatric ‘Reform’ in Greece: The Crime with the Cloak of Innocence],” *Σύγχρονα θέματα [Contemporary Issues]*, no. 19 (1983): 61–70; Michalis Madianos, “Το Κέντρο Κοινοτικής Ψυχικής Υγιεινής στην Ελλάδα σήμερα: η πρώτη εμπειρία στην Καισαριανή-Βύρωνα [The Center of Community Mental Health in Greece Today: The First Experience in Kaisariani-Vyronas],” in *Greek-French Symposium of Social Psychiatry*, 197–208; Panagiotis Sakellaropoulos et al., “Κινητή Μονάδα Ψυχιατρικής Περίθαλψης Υπαίθρου (ΚΜΨΠΥ) [Mobile Unit of Psychiatric Care of Rural Areas],” in *Greek-French Symposium of Social Psychiatry*, 241–244; Kostas Mpairaktaris, *Ψυχική υγεία και κοινωνική παρέμβαση. Εμπειρίες, συστήματα, πολιτικές [Mental Health and Social Intervention. Experiences, Systems, Policies]* (Athens: Enallaktikes Ekdoseis, 1994), 112–135.

37 The Association against Prejudice on Mental Disorder (Continuity) was founded in 1984 by patients who participated in these professional programs, and was supported by the Scientific University Institute for Mental Health in Athens. Among its aims were patients’ empowerment and self-help and the promotion of their rights. Ioannis Cheilakos, “Η δημιουργία και η λειτουργία συλλόγων αυτοβοήθειας και αυτοδιαχείρισης ασθενών [The Creation and Operation of Associations of Self-Help and Self-Management of Patients],” in *Ψυχική υγεία. Σύγχρονες προσεγγίσεις-προβληματισμοί [Mental Health. Contemporary Approaches - Concerns]*, ed. Eleni Grammatikopoulou (Athens: National Institute of Research, 2011), 103–108.

38 Manolis Tzanakis, *Πέραν του ασύλου: Η κοινοτική ψυχιατρική και το ζήτημα του υποκειμένου [Beyond the Asylum: Community Psychiatry and the Issue of the Subject]* (Thessaloniki: Koinos Topos Psychiatrikis, Nevroepistimon ke Epistimon tou Anthropou, 2008), 110–111.

39 Theodoros Megaloeconomou, “Μια ζωντανή αμφισβήτηση της κλασικής ψυχιατρικής [A Living Contestation of Classical Psychiatry],” in *Λέρος. Μια ζωντανή αμφισβήτηση της κλασικής ψυχιατρικής [Leros. A Living Contestation of Classical Psychiatry]*, ed. Theodoros Megaloeconomou (Athens: Agra, 2016), 23–42; Mpairaktaris, *Mental Health and Social Intervention*, 103–104.

40 By the end of the 1970s, the major works of antipsychiatry and other radical psychiatric literature of the 1960s and 1970s had been translated and published in Greek. In addition, a magazine of anti-psychiatric orientation, *Η τρέλλα* [*Madness*], published three issues between 1979 and 1981, and presentations of anti-psychiatric thought appeared in other magazines of the late 1970s and the 1980s, including the literary magazine *Διαβάζω* [*I* r*ead]* and the left underground magazines *Ιδεοδρόμιο* [*Road of Ideas*] and *Ιανός* [Janus]. A fervent supporter of radical psychiatry was the psychiatrist Kleanthis Grivas. See for example, Kleanthis Grivas, *Ψυχιατρικός ολοκληρωτισμός. Ιστορική και κοινωνική θεώρηση της ψυχιατρικής [Psychiatric Totalitarianism. Historical and Social View of Psychiatry]* (Thessaloniki: Ianos, 1985).

41 Theodoros Megaloeconomou, “Franco Basaglia’s Influence on the Greek Mental Health System,” in *Basaglia’s International* Legacy, 161–174. There was a growing number of Greek students in Italy (Kornetis, *Children of the Dictatorship*, 146–151) and since the 1980s Greek mental health professionals exchanged visits with colleagues from Trieste, an internationally acclaimed center of psychiatric reform (Theodoros Megaloeconomou, “Ιστορία των παρεμβάσεων αποδρυματοποίησης στο Κρατικό Θεραπευτήριο Λέρου [History of the Interventions of Deinstitutionalization to the State Mental Hospital of Leros],” in *Leros. A Living Contestation of Classical Psychiatry*, 115–148).

42 Ioannis Tsegos, “Ιδεολογικοπολιτικοί προβληματισμοί περί την ψυχιατρική και την ψυχοθεραπεία κατά τη Μεταπολίτευση [Ideological and political concerns about psychiatry and psychotherapy during the post-dictatorship period],” in *Ανοικτό Ψυχοθεραπευτικό Κέντρο (1980-2007). Δραστηριότητες και ιδιοτροπίες [Open Psychotherapy Center (1980-2007). Activities and Peculiarities]*, ed. Ioannis Tsegos et al. (Athens: Enallaktikes Ekdoseis, 2007), 19–28, 27.

43 Fotini Tsalikoglou, “Οι μυθικές διαστάσεις της παραφροσύνης. Για την αντιψυχιατρική [The Mythical Dimensions of Madness. On Antipsychiatry],” *Διαβάζω [I Read]*, no. 65 (1983): 52–58.

44 “Αισώπου μύθοι και το πανόραμα της νέας ψυχιατρικής [Myths of Aesop and the Panorama of the New Psychiatry],” *Η τρέλλα [Madness]*, no. 2 (1980): 6–9.

45 Ioannis Tsegos, “Επίμετρο: η ψυχιατρική μεταρρύθμιση [Addendum: Psychiatric Reform],” in *Open Psychotherapy Center (1980-2007)*, 28–31, 28.

46 Tzanakis, *Beyond the Asylum*, 110–111.

47 For examples of patients’ participation, self-representation, and organization during this period, see Fafaliou, *Iera Odos 343*; Maria Fafaliou and Koralia Sotiriadou, ed., *Το ρούχο της αθωότητας. Ποίηση ανθρώπων με ψυχιατρική εμπειρία [The Cloak of Innocence. Poetry by People with Psychiatric Experience]* (Athens: Estia, 2013); Maria Kalogeropoulou, “Η συμβολή των οργανώσεων χρηστών των υπηρεσιών υγείας ψυχικής υγείας στην καταπολέμηση του στίγματος [The Contribution of the Organizations of Users of Mental Health Services to the Fight against Stigma]” (University of Athens, Department of Nursing, 2005); Center of Mental Health of Egaleo - Network of Services for Rehabilitation “Make it With Us” and Kalidoskopio, “Η αυτοεκπροσώπηση πραγματικά δουλεύει [Self-Representation Really Works]” (Day Conference Proceedings, Athens, 2001).

48 “Σύνθεση και συζήτηση στα θέματα της ημέρας [Synthesis and Discussion on the Day's Topics],” in *Greek-French Symposium of Social Psychiatry*, 441–456, 452-455. In this Symposium which took place in 1981 and represented new trends in mental health care, reservations about patients’ self-representation were expressed by progressive psychiatrists who had started working in the 1940s and 1950s, such as the psychiatrists Georgios Lyketsos, Efstathios Lymperakis, and Serge Lebovici. They believed, however, that with professional help, patients did benefit from self-help groups and therapeutic clubs.

49 “Κίνηση για τα Δικαιώματα των ‘Ψυχασθενών’ – διακήρυξη [Motion for the Rights of the ’Mentally Ill’ - Declaration],” *Freedom Is Therapeutic*, no. 1 (1982): 4–5.

50 L., “Σχιζοφρενικά, παρανοκά και άλλα [Schizophrenic, Paranoid and Other Topics],” *Freedom Is Therapeutic*, no. 1 (1982): 6.

51 Hatzidaki, “Psychiatric ‘Care’ and Psychiatric ‘Reform’ in Greece,” 63.

52 I.F.A., “Η πραγματική βάση της ιατρικής είναι η αγάπη [The True Basis of Medicine Is Love],” *Freedom Is Therapeutic*, no. 1 (1982): 24–27; “Motion for the Rights of the ’Mentally Ill’ - Declaration.”

53 “Τα αδημοσίευτα [The Unpublished],” *Freedom Is Therapeutic*, no. 2 (1983): 18; “Καταγγελία [Denunciation],” *Freedom Is Therapeutic*, no. 9 (1989): 30; P. G., “Τα διαφημιστικά των ψυχοφαρμάκων [The Advertisements of Psychiatric Drugs],” *Freedom Is Therapeutic*, no. 1 (1982): 8–11; John Friedberg, “Θεραπεία με ηλεκτροσόκ. Ας σταματήσουμε να ανατινάζουμε το μυαλό [Electroshock Treatment. Let’s Stop Blowing up the Mind],” *Freedom Is Therapeutic*, no. 3–4 (1984): 43–55.

54 See for example, Hatzidaki, “Handling the ‘Mentally Ill’”; “Εθνικό Σύστημα Υγείας: μετωνυμίες και αποσιωπήσεις [National Health System: Metonymies and Concealments],” *Freedom Is Therapeutic*, no. 2 (1983): 10–11; Varvara Delivoria, “Η ψυχιατρική καταστολή και τα δικαιώματα των ‘ψυχασθενών’ πολιτών [Psychiatric Repression and the Rights of the ‘Mentally Ill’ Citizens],” *Freedom Is Therapeutic*, no. 5–6 (1985): 84–86; “Ανοιχτή επιστολή στον υπ. Υγείας κ. Φλώρο [Open Letter to the Minister of Health Mr. Floro],” *Freedom Is Therapeutic*, no. 7–8 (1988): 37–38.

55 P. G., “Η ψυχιατρικοποίηση της είδησης από τον ελληνικό τύπο [The Psychiatrization of the News by the Greek Press],” *Freedom Is Therapeutic*, no. 1 (1982): 14–15; “Μικρό αφιέρωμα ΕΣΥ και ΕΜΕΙΣ [Small Cover Story on the National Health System and Us],” *Freedom Is Therapeutic*, no. 5–6 (1985): 50–60.

56 Papadogiannis, "From Coherence to Fragments"; Theodosis Gkeltis, “Το ομοφυλόφιλο κίνημα στην Ελλάδα της μεταπολίτευσης: η ανάδυση ενός νέου συλλογικού υποκειμένου [The Gay Movement in Post-Dictatorship Greece: The Emergence of a New Collective Subject],” radio program Η Ιστορία στο Κόκκινο [History in Red], 25 February 2018.

57
*Freedom Is Therapeutic*, no. 1 (1982): 2; *Freedom Is Therapeutic*, no. 2 (1983): 65. In either case, the anonymity of many of the authors makes it difficult to know more about their identity and more generally about the identity of the Motion’s members. More is known about members who were mental health professionals (see below).

58 Varvara Delivoria, “Η ψυχιατρική βία είναι πολιτική βία. Η ΚΔ ‘Ψ’ στο φεστιβάλ Αυγής-Θουρίου [Psychiatric Violence is Political Violence. The Motion for the Rights of the ‘Mentally Ill’ in the Festival Avgis-Thouriou],” *Freedom Is Therapeutic*, no. 2 (1983): 4–5; “Κίνηση για τα Δικαιώματα των ‘Ψυχασθενών’ [Motion for the Rights of the ‘Mentally Ill’],” *Freedom Is Therapeutic*, no. 1 (1982): 28–29; D. Ch. D., “Και τώρα τι θα γίνουμε χωρίς κλινικές; [And Now, What Will Become of Us without Clinics?],” *Freedom Is Therapeutic*, no. 1 (1982): 12–13; “Χαιρετισμός της ΚΔ ‘Ψ’ στη συνέντευξη Τύπου που έδωσε το ΑΜΦΙ-ΑΚΟΕ στις 5/4/82 [Salutation of the Motion for the Rights of the ‘Mentally Ill’ in the Press Conference of AMFI-AKOE on 5 April 1982],” *Freedom Is Therapeutic*, no. 1 (1982): 18; “Small Cover Story on the National Health System and Us.”

59 “Small Cover Story on the National Health System and Us”; "Motion for the Rights of the ‘Mentally Ill'"; Giorgos Koukis, “Απ’ την πλευρά των ‘άλλων’ (Συνειρμοί και προσεγγίσεις γύρω από την έννοια ’ρατσισμός’) [From the Side of the ‘Others’ (Associations and Approaches of the Concept ’Racism’)],” *Freedom Is Therapeutic*, no. 9 (1989): 18–19.

60 Lampros Iosif, “Μια στο καρφί και μια στο πέταλο [One Hit after the Other],” *Freedom Is Therapeutic*, no. 7–8 (1988): 43–47, 47.

61 For instance, the Groupe Information Asiles, the first French mental patients’ organization, founded in 1972, had the slogan “We all have a psychiatric past or future.” For a history of the GIA, see “Aperçu historique du GIA de 1972 à 1992 (entretien avec Philippe Bernardet),” in *Pour en finir avec la psychiatrie. Des patients témoignent*, ed. Nicole Maillard-Déchenans (Saint Georges d’Oléron: Editions Libertaires, 2008), 239–280.

62 Natasa K., “Οι τίτλοι του τρόμου [The Headlines of Terror],” *Freedom Is Therapeutic*, no. 2 (1983): 40–41. The Mental Hospital of Leros often made the news during the 1980s. Τhe Motion was also writing about it and collaborated with the “Group of the Doctors who had worked in Leros,” a dynamic group that had publicized the situation in the hospital in Greece and abroad, asking for radical change.

63 D. Ch. D., "And Now, What Will Become of Us without Clinics?"; Hatzidaki, "Handling the ‘Mentally Ill’"; “Για τα ναρκωτικά ή ψυχίατροι και αστυνόμοι [On Drugs or Psychiatrists and Policemen],” *Freedom Is Therapeutic*, no. 2 (1983): 30–39; “Open Letter to the Minister of Health Mr. Floro.”

64 “Το ιταλικό πείραμα [The Italian Experiment],” *Freedom Is Therapeutic*, no. 10 (1993): 20–25; Giorgos Koukis, “Ψυχιατρείο – ένας μύθος ελάχιστα ρομαντικός [Mental Hospital - A Barely Romantic Myth],” *Freedom Is Therapeutic*, no. 10 (1993): 26–28.

65 In addition, in the 1990s the Motion used “Freedom Is Therapeutic” as its name, as it was thought that the term “mentally ill” in the original appellation did not sound well. Natasa Karapostoli was a steady member of the Motion and, for many years, on its administration board. She had obtained her degree in occupational therapy from the Department of Physical Therapy, Center of Higher Technical and Professional Education (KATEE) of Athens in 1983 and was later trained in psychodrama, sociotherapy, and family therapy in the Open Psychotherapy Center. Natasa Karapostoli, interview with the author, 30 November 2019. Those who joined the Motion in the 1990s refer to it exclusively as the Motion Freedom Is Therapeutic. Dionysis Perros was a patient of the Open Psychotherapy Center in the 1990s and a member of the Motion and its successive organization, Antistigma. During his student years he had been an active member of Communist Youth of Greece, which was affiliated with the Communist Party of Greece. Dionysis Perros, interview with the author, 13 June 2020.

66 Karapostoli, “’Anamnesis,’” 12.

67 F. K. T., “Thomas Szasz, *Η Βιομηχανία της Τρέλας* [Review of Thomas Szasz, *The Manufacture of Madness*],” *Freedom Is Therapeutic*, no. 2 (1983): 59–60. The article was anonymous, and it cannot be ascertained if it was written by a professional or not.

68 Hatzidaki, “Handling the ‘Mentally Ill.’”

69 Karapostoli, interview with the author.

70 “Motion for the Rights of the ‘Mentally Ill.’”

71 “Οι μεγαλύτεροι ψυχίατροι του κόσμου [The Greatest Psychiatrists of the World],” *Freedom Is Therapeutic*, no. 3–4 (1984): 12–13; Lia Sotiriou, “Συνέχεια… παρόντες [Constantly… Present],” *Freedom Is Therapeutic*, no. 5–6 (1985): 30–31; Z. V., “Οι αδούλωτοι αγώνες ενός διευθυντή ψυχιατρείου [The Indomitable Struggles of a Mental Hospital Director],” *Freedom Is Therapeutic*, no. 2 (1983): 15.

72 "The Greatest Psychiatrists of the World"; G. P., “Γιατροί και βασανιστήρια ή ένας επιδέξιος ελιγμός [Doctors and Torture or a Skillful Maneuver],” *Freedom Is Therapeutic*, no. 5–6 (1985): 21.

73 For example, the psychiatrists Ioannis Tsegos and Athanasia Kakouri-Bassea, and the occupational therapist Natasa Karapostoli, were among the Center’s staff who were long-standing members of the Motion. Tsegos was trained in group analysis in London, England. He introduced the first analytic groups in Greece when he returned from London in 1978. Kakouri-Bassea had studied in the late 1970s in Rome, Italy, where she was influenced by social and democratic psychiatry. Although psychiatry maintained its dominance in mental health care and reform during the 1980s, the discipline of psychology was getting more established. It is indicative that in 1984 the first university psychology department was founded at the University of Crete, and a psychologist, Kostas Mpairaktaris, furthered the psychiatric reform in Thessaloniki (Mpairaktaris, *Mental Health and Social Intervention*, 112-135). Finally, it should be noted that the first president of the Motion, Rena Hatzidaki, was neither psychiatrist or psychologist. Α poet, translator, and editor who had been imprisoned during the dictatorship, she underwent psychotherapy in the Center and was trained and worked there as a psychotherapist before setting up her own practice in 1987.

74 Athanasia Kakouri-Bassea and Dimitris Moschonas, “Γενική θεώρηση [General View],” in *Open Psychotherapy Center (1980-2007)*, 35–41.

75 Ioannis Tsegos, interview with the author, 27 November 2020. Tsegos explained the Motion's will for independence in terms of its move from the building where it was initially based, very close to the Center, to a farther away one in 1988. During this period and until the early 1990s, professionals from the Wilhelm Reich Center of Athens were playing an increasingly important role in the Motion. Perros, interview with the author, and articles by the psychiatrist Georgios Koukis, of the Wilhelm Reich Center, in *Freedom Is Therapeutic*, for example, Koukis, "From the Side of the ‘Others’"; Koukis, “Mental Hospital - A Barely Romantic Myth.”

76 According to a retrospective of the other patient organization founded in the 1980s, the Association Against Prejudice on Mental Disorder (Continuity), this organization had been criticized for being less challenging and demanding because professionals supported it. However, this support was deemed essential for the continuation of the Association’s work for twenty five years. *Πρακτικές ενδυνάμωσης στην κοινωνική πολιτική. Δουλεύοντας μαζί στην εκπαίδευση ψυχικής υγείας [Practices of Empowerment in Social Policy. Working together in Mental Health Education]* (Athens, 2009), 47–48.

77 For example, the magazine published a photograph from a protest against ECT in 1975 by the Network Against Psychiatric Assault – a radical organization from the USA – and a caricature from the magazine of the less radical British organization MIND. *Freedom Is Therapeutic*, no. 3-4 (1984): 54; *Freedom Is Therapeutic*, no. 1 (1982): 7.

78 “Αλληλογραφία… και με το εξωτερικό [Correspondence … and from Abroad],” *Freedom Is Therapeutic*, no. 5–6 (1985): 95. The Motion noted that it had established a correspondence with organizations from Cyprus, England, Sweden, Finland, Belgium, and Canada. “Νέα απ’το εξωτερικό [News from Abroad],” *Freedom Is Therapeutic*, no. 2 (1983): 63.

79 “Συμμετοχή της ΚΔΨ σε ευρωπακά συνέδρια [Participation of the Motion for the Rights of the Mentally Ill in European Conferences],” *Freedom Is Therapeutic*, no. 9 (1989): 2–3; http://enusp.org/who-we-are/.

80 “Small Cover Story on the National Health System and Us.”

81 G. K., “Πόλεμος πατήρ πάντων [War, Father of Everything],” *Freedom Is Therapeutic*, no. 7–8 (1988): 19–23.

82 Delivoria, “Psychiatric Repression,” 86.

83 Hatzidaki, “Handling the ‘Mentally Ill,’” 3.

84 Ibid.

85 “Motion for the Rights of the ’Mentally Ill’ - Declaration.”

86 Kyriakos Theodorakakos, “Μνήμες Μαíου συνεδριακές [Conference Memories of May],” *Freedom Is Therapeutic*, no. 7–8 (1988): 5–6.

87 Kornetis, “Transitions, Collective Memory and Public History.”

88 “Προκήρυξη που κυκλοφόρησε η ΚΔ ‘Ψ’ στις 15 του Νοέμβρη 1982 [Proclamation Distributed by the Motion for the Rights of the ‘Mentally Ill’ on November 15, 1982],” *Freedom Is Therapeutic*, no. 1 (1982): 21.

89 Papadogiannis, “From Coherence to Fragments”; Glystras, “The ‘Other’ Left,” 25, 127, 130, 193–194, 200.

90 “Ψήφισμα. Κίνηση για τα Δικαιώματα των ‘Ψυχασθενών’ [Vote. Motion for the Rights of the ’Mentally Ill’],” *Freedom Is Therapeutic*, no. 5–6 (1985): 35; "Salutation"; Iosif, “One Hit after the Other.”

91 “Η ΚΔ ‘Ψ’ και τα άλλα κοινωνικά κινήματα [The Motion for the Rights of the ‘Mentally Ill’ and the Other Social Movements],” *Freedom Is Therapeutic*, no. 1 (1982): 19. During this period there was considerable communication and overlap among different social movements – feminist, gay, environment, and prison reform – as the same people participated in different organizations and mobilizations. Gkeltis, “The Gay Movement in Post-Dictatorship Greece.”

92 Vangelis Karamanolakis, discussion with the author, 20 December 2019.

93 Delivoria, “Psychiatric Violence is Political Violence.”

94 "The Unpublished"; “Denunciation.”

95 However, this project had also the aim to put pressure on institutions against which accusations were made. Giorgos Koukis, “Αν το πάθος σας είναι οι εξερευνήσεις… [If your Passion Is to Explore…],” *Freedom Is Therapeutic*, no. 10 (1993): 6.

96 Karapostoli, “‘Anamnesis.’”

97 Nikolaos Papadogiannis, “Νεανική πολιτικοποίηση και ‘πολιτισμός’ στα πρώτα χρόνια της μεταπολίτευσης [Youthful Politicization and ‘Culture’ in the First Years of the Post-Dictatorship Period],” in *The Post-Dictatorship Period*, 133–150; Tzanakis, *Beyond the Asylum*, 110–111.

98 Theodorakakos, “Conference Memories of May.”

99 Perros, interview with the author; Dimitris Moschonas, personal communication with the author, 24 June 2020. Another organization, “Antistigma. Network for the Rights and the Violations in the Field of Mental Health,” succeeded the Motion in 2007. Antistigma was active until the early 2010s, and was not a union, like the Motion, but a non-profit civil partnership, like the Open Psychotherapy Center. Dimitris Moschonas is a psychiatrist and psychotherapist who cooperated with the Center and was active in both the Motion and Antistigma.

100 Karapostoli, “‘Anamnesis,’” 13.

101 Delivoria, “Psychiatric Violence is Political Violence.”

102 Rabeharisoa, “From Representation to Mediation.”

